# Prognostic value of a 25-gene assay in patients with gastric cancer after curative resection

**DOI:** 10.1038/s41598-017-07604-y

**Published:** 2017-08-08

**Authors:** Xiaohong Wang, Yiqiang Liu, Zhaojian Niu, Runjia Fu, Yongning Jia, Li Zhang, Duanfang Shao, Hong Du, Ying Hu, Xiaofang Xing, Xiaojing Cheng, Lin Li, Ting Guo, Ziyu Li, Qunsheng Ji, Lianhai Zhang, Jiafu Ji

**Affiliations:** 1 Key laboratory of Carcinogenesis and Translational Research (Ministry of Education), Central Biobank Facility, Peking University Cancer Hospital and Institute, Beijing, China; 2 Department of Pathology, Peking University Cancer Hospital and Institute, Beijing, China; 3 Department of Surgery, Peking University Cancer Hospital and Institute, Beijing, China; 4 Department of General Surgery, Affiliated Hospital of Qingdao University, Shandong Province, China; 5 Asia & Emerging Markets Innovative Medicine, AstraZeneca R&D, Shanghai, China

## Abstract

This study aimed to develop and validate a practical, reliable assay for prognosis and chemotherapy benefit prediction compared with conventional staging in Gastric cancer (GC). Twenty-three candidate genes with significant correlation between quantitative hybridization and microarray results plus 2 reference genes were selected to form a 25-gene prognostic classifier, which can classify patients into 3 distinct groups of different risk of mortality obtained by analyzing microarray data from 78 frozen tumor specimens. The 25-gene assay was associated with overall survival in both training (*P* = 0.017) and testing cohort (*P* = 0.005) (462 formalin-fixed paraffin-embedded samples). The risk prediction in stages I + II is significantly better than that in stages III. Analysis demonstrated that this 25-gene signature is an independent prognostic predictor and show higher prognostic accuracy than conventional TNM staging in early stage patients. Moreover, only high-risk patients in stage I + II were found benefit from adjuvant chemotherapy (*P* = 0.043), while low-risk patients in stage III were not found benefit from adjuvant chemotherapy. In conclusion, our results suggest that this 25-gene assay can reliably identify patients with different risk for mortality after surgery, especially for stage I + II patients, and might be able to predict patients who benefit from chemotherapy.

## Introduction

Gastric cancer (GC) is an aggressive malignancy with high rate of recurrence in patients even undergoing curative resection. Post-operative management depends on accurate prognostic staging to identify the individuals at high risk for relapse. Modest efficacy and considerable toxicities associated with adjuvant chemotherapy. Many factors can affect the prognosis of GC patients, such as TNM stage, while this has limited ability to stratify patients according to their likely outcome^1^. Moreover, there also have great variation in the histological appearance of GC^2^. So the current evaluation system for GC is insufficient for predicting the outcome of GC treatment. A more precise staging test would give clinicians the ability to identify patients with statistically heterogeneous outcomes from within otherwise homogeneous clinical groups.

GC is a multistage process involving the accumulation of genetic and epigenetic alterations which may be the important prognostic factors. Gene expression profiling is useful for classifying tumors for various types of cancer patients, and can predict the prognosis for patients with various types of cancer^3, 4^. However, most prognostic prediction models focused on breast cancer and lung cancer^5, 6^, only a few studies on GC prognosis^7, 8^. The profiling genes selected in GC studies have varied considerably. Gene expression profiles were also different according to the microarray platform and the analytic strategy used^9–14^.

In certain research situations, performing such analyses on archival formalin fixed paraffin-embedded (FFPE) surgical specimens would be advantageous as large libraries of such specimens with long-term follow-up data are widely available. To systematically validate the microarray results, branched DNA signal amplification (Quantigene Plex, QGP), quantitative hybridization assays, which can measure 3 to 80 target RNAs per well with unparalleled accuracy and precision, was used^15, 16^. In this study, we developed and validated a prognosis-associated 25-gene assay using QGP on FFPE samples. And we also investigated the chemotherapy benefit in different risk group.

## Results

### Screening of candidate biomarkers by microarray and establishment of a 31-gene prognostic algorithm

Detailed clinicopathological characteristics of the selecting, training, and testing sets were shown in Table 1. For initial screening of candidate biomarkers by microarray profiling, we selected 78 patients with qualified frozen tissues in the first batch.

**Table 1:** Clinical and pathological characteristics of patients in three cohorts.

	Selecting Cohort	Training Cohort^*^	Test Cohort
	N = 78	N = 102	N = 360
Age at resection (years; mean^32^)	61.1 ± 9.83	61.38 ± 9.51	58.97 ± 12.26
Sex			
Male	58 (74.36%)	79 (77.45%)	246 (68.33%)
Female	20 (25.64%)	23 (22.55%)	114 (31.67%)
Differentiation			
Well-Moderately differentiated	15 (19.23%)	11 (10.78%)	108 (30%)
Poorly differentiated	63 (80.77%)	91 (89.22%)	239 (66.39%)
Undetermined	0	0	13 (3.61%)
Lauren Subtype			
Diffuse Type	26 (33.33%)	35 (34.31%)	75 (20.83%)
Intestinal Type	38 (48.72%)	42 (41.18%)	242 (67.22%)
Mixed Type	14 (17.95%)	25 (24.51%)	43 (11.94%)
Location			
Cardia	27 (34.62%)	30 (29.41%)	105 (29.17%)
Non-cardia	51 (65.38%)	72 (70.59%)	255 (70.83%)
TNM Stage			
I	3 (3.85%)	6 (5.88%)	36 (10%)
II	17 (21.79%)	23 (22.55%)	90 (25%)
III	48 (61.54%)	73 (71. 57%)	234 (68.82%)
IV	10 (12.82%)	0	0
T Stage			
1	0 (0%)	1 (0%)	20 (5.11%)
2	7 (8.97%)	7 (9.84%)	40 (10.46%)
3	26 (33.33%)	45 (31.15%)	101 (30.9%)
4	45 (57.69%)	49 (59.02%)	199 (53.53%)
N Stage			
0	14 (17.95%)	19 (18.63%)	96 (26.67%)
1	11 (14.10%)	15 (14.71%)	53 (14.72%)
2	19 (24.36%)	28 (27.45%)	78 (21.67%)
3	34 (43.59%)	40 (39.22%)	133 (36.94%)
M Stage			
0	68 (87.18%)	102 (100%)	360 (100%)
1	10 (12.82%)	0	0
Vascular invasion			
V(−)	31 (39.74%)	43 (42.16%)	179 (49.72%)
V(+)	42 (53.85%)	55 (53.92%)	181 (50.28%)
Not recorded*	5 (6.41%)	4 (3.92%)	0

*There is 51 cases overlapped in selecting cohort and training cohort.

On Affymetrix microarray analysis of tumors from the 78 cancer and 24 matched adjacent non-cancerous gastric mucosa, 2880 genes showed significantly differential expression between the GC tissues and adjacent non-cancerous gastric mucosa. We used a Cox proportional hazards modeling as the main analytical test used to develop the prognostic algorithm (to build a prognostic classifier), which selected31 target genes (Table 2) in the 78-patient selecting cohort. Among them, 14 genes were correlated with patient prognosis analyzed by hazard ratios from univariate Cox regression, including 6 protective genes (*XAF1*, *IFITM1*, *NCOA7*, *GZF1*, *APAF1*, and *TCF7L2*, with hazard ratio less than 1), and 8 risk genes (*DYRK2, UBA2*, *EPHB2*, *PDCD5*, *FADD*, *MARCKS*, *B3GALT6*, and *ITCH*, with hazard ratio more than 1), while 17 genes were related to classical oncogenic pathways or potential therapeutic targets in GC from previous publication, including *MMP2*, *EGFR*, *MMP7*, *MET*, *ERBB2*, *CDK1*, *CDK6*, *SRC*, *IGF1R*, *CDK4*, *PDGFRB*, *ERBB3*, *PARP1*, *FRAP1*, *CDK3*, *FLT4*, and *KDR*.

**Table 2:** Representative amplified genomic loci and genes by microarray analysis.

gene	Gene name	Chromosome	Reference sequence	Protein location	Relevant biological functions and pathways	Role in algorithm	P^*^ value	Correlation Coefficient	P^#^ value	HR(95%CI)
**Survival associated genes from Chip results**										
XAF1	XIAP associated factor 1	17p13.1	NM_017523	cytoplasm, nucleus	Affect the progress of the apoptosis signaling pathway	prognosis	0	0.583	0	0.564 (0.424–0.752)
IFITM1	interferon induced transmembrane protein 1	11p15.5	NM_003641	membrane, plasma membrane	Influence cell invasion	prognosis	0	0.74	0.001	0.639 (0.489–0.834)
DYRK2	dual-specificity tyrosine-(Y)-phosphorylation regulated kinase 2	12q15	NM_003583	cytoplasm, nucleus,membrane	Tyrosine autophosphorylation and catalyzed phosphorylation of histones H3 and H2B	prognosis	0	0.53	0.001	2.078 (1.347–3.205)
NCOA7	nuclear receptor coactivator 7	6q22.32	NM_181782	intracellular, nucleus	Cell wall macromolecule catabolic process, positive regulation of transcription	prognosis	0	0.581	0.003	0.514 (0.330–0.801)
UBA2	ubiquitin-like modifier activating enzyme 2	19q12	NM_005499	cytoplasm, nucleoplasm	SUMO-activating enzyme for the sumoylation of proteins	prognosis	0	0.577	0.011	2.064 (1.184–3.599)
EPHB2	EPH receptor B2	1p36.1-p35	NM_004442	membrane	Angiogenesis, axon guidance	prognosis	0	0.56	0.013	1.593 (1.101–2.305)
PDCD5	programmed cell death 5	19q13.11	NM_004708	cytoplasm, nucleus	Apoptotic process	prognosis	0	0.451	0.02	1.756 (1.094–2.820)
FADD	Fas (TNFRSF6)-associated via death domain	11q13.3	NM_003824	membrane, plasma membrane	Apoptotic signaling pathway,TRIF-dependent toll-like receptor signaling pathway	prognosis	0	0.665	0.033	1.579 (1.038–2.403)
B3GALT6	UDP-Gal:betaGal beta 1,3-galactosyltransferase polypeptide 6	1p36.33	NM_080605	Golgi membrane	Carbohydrate metabolic process	prognosis	0	0.595	0	2.256 (1.484–3.428)
MARCKS	myristoylated alanine-rich protein kinase C substrate	6q22.2	NM_002356	plasma membrane	Energy reserve metabolic process	prognosis	0.001	0.409	0.015	1.985 (1.141–3.452)
GZF1	GDNF-inducible zinc finger protein 1	20p11.21	NM_022482	nucleolus	Negative regulation of transcription, DNA-templated	prognosis	0.011	0.323	0	0.564 (0.424–0.752)
APAF1	apoptotic peptidase activating factor 1	12q23	NM_013229	cytoplasm, nucleus	Activation of cysteine-type endopeptidase activity involved in apoptotic process	prognosis	0.029	0.28	0.001	0.279 (0.127–0.610)
ITCH	itchy E3 ubiquitin protein ligase	20q11.22	NM_031483	cytoplasm, nucleus, membrane	Notch signaling pathway, apoptotic process, inflammatory response	prognosis	0.224	0.158	0.005	2.475 (1.311–4.671)
TCF7L2	transcription factor 7-like 2	10q25.3	NM_030756	cytoplasm	Canonical Wnt receptor signaling pathway, fat cell differentiation	prognosis	0.645	0.061	0	0.209 (0.112–0.389)
**GC therapeutic targets**										
EGFR	epidermal growth factor receptor	7p12	NM_005228	membrane, cytoplasm	MAP kinase kinase kinase activity, cell proliferation	prognosis	0	0.691		
MMP7	matrix metallopeptidase 7	11q21-q22	NM_002423	plasma membrane, extracellular region	Metalloendopeptidase activity, regulation of cell proliferation	prognosis	0	0.656		
MET	met proto-oncogene	7q31	NM_000245	plasma membrane	Activation of MAPK activity, cell proliferation	prognosis	0	0.534		
ERBB2	v-erb-b2 avian erythroblastic leukemia viral oncogene homolog 2	17q12	NM_004448	plasma membrane	Cell proliferation, ATP binding	prognosis	0	0.743		
CDK1	cyclin-dependent kinase 1	10q21.1	NM_001786	cytoplasm, nucleus	DNA repair, DNA replication	prognosis	0	0.498		
CDK6	cyclin-dependent kinase 6	7q21-q22	NM_001259	cytoplasm, nucleus	G1/S transition of mitotic cell cycle	prognosis	0	0.661		
IGF1R	insulin-like growth factor 1 receptor	15q26.3	NM_000875	plasma membrane	Inactivation of MAPKK activity, insulin receptor signaling pathway	prognosis	0	0.486		
CDK4	cyclin-dependent kinase 4	12q14	NM_001259	cytoplasm, nucleus	G2/S transition of mitotic cell cycle, cell division	prognosis	0	0.561		
SRC	v-src avian sarcoma	20q12-q13	NM_005417	cytoplasm, nucleus	Ras protein signal transduction	prognosis	0.001	0.403		
KDR	kinase insert domain receptor	4q11-q12	NM_002253	plasma membrane	Angiogenesis, endothelial cell differentiation	prognosis	0.002	0.397		
MMP2	matrix metallopeptidase 2	16q13-q21	NM_004530	plasma membrane, extracellular region	Regulation of vascularization and the inflammatory response	prognosis	0.086	0.221		
PDGFRB	platelet-derived growth factor receptor, beta polypeptide	5q33.1	NM_002609	plasma membrane	G-protein coupled receptor signaling pathway, cell migration	prognosis	0.148	0.187		
ERBB3	v-erb-b2 avian erythroblastic leukemia viral oncogene homolog 3	12q13	NM_001982	plasma membrane	Growth factor binding, negative regulation of cell adhesion	prognosis	0.154	0.185		
PARP1	poly (ADP-ribose) polymerase 1	1q41-q42	NM_001618	nucleolus	DNA damage response, detection of DNA damage	prognosis	0.458	0.097		
FRAP1	mechanistic target of rapamycin	1p36.2	NM_004958	membrane, cytoplasm	TOR signaling, ATP binding, drug binding, cell growth	prognosis	0.738	0.044		
FLT4	fms-related tyrosine kinase 4	5q35.3	NM_001258	cytoplasm, plasma membrane	Blood vessel morphogenesis, negative regulation of apoptotic process	prognosis	0.745	−0.043		
CDK3	cyclin-dependent kinase 3	17q25.1	NM_002021	cytoplasm, nucleus	Cyclin-dependent protein serine/threonine kinase activity, G0 to G1 transition, cell proliferation	prognosis	0.892	0.018		
**Reference genes**										
TBP	TATA box binding protein	6q27	NM_003194	nucleolus	Transcription initiation; RNA elongation; transcription	Reference				
PGK1	phosphoglycerate kinase 1	Xq13.3	NM_000291	cytoplasm	ATP binding; phosphoglycerate kinase activity; carbohydrate metabolic process	Reference				

P^*^ values for the correlation coefficients were estimated by Pearson correlation test.

P^#^ values for the hazard ratios were estimated by univariate Cox regression analysis of the microarray data.

**Figure 1:**
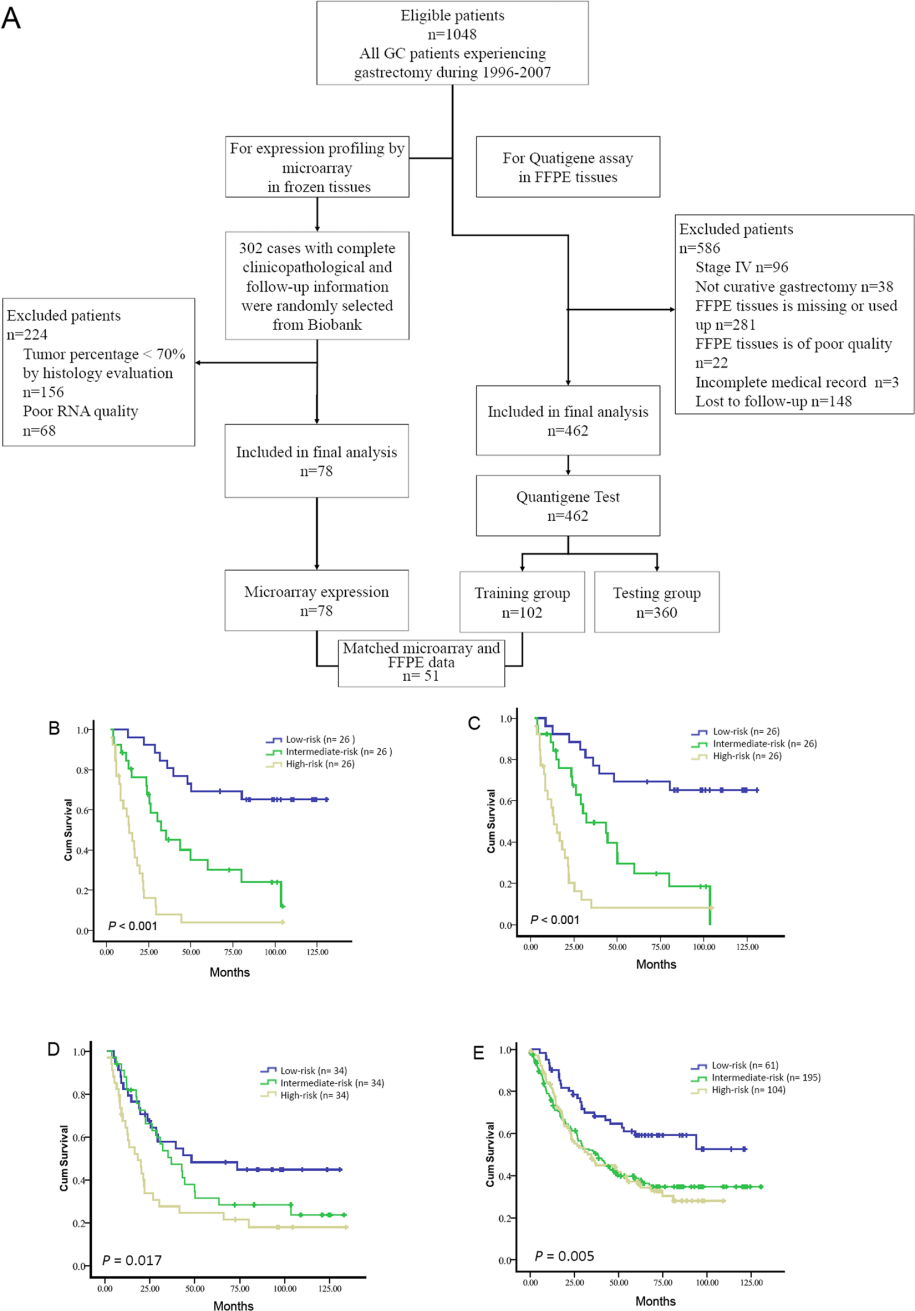
Study design and the combined gene signature and survival in GC. Panel A showed the study design. Kaplan–Meier survival curve estimated the overall survival according to the 31 gene microarray signature (**B**) and 25-gene microarray signature^33^. Kaplan-Meier survival curves for training and testing cohort according to 25-gene signature were showed in Panel D and E.

We then derived a formula (Supplementary materials and methods) to calculate the risk score for their risk of mortality for every patient based on their individual 31-gene expression levels. Those GC patients with a high-risk thirty-one gene signature had a shorter median overall survival than the patients with intermediate-risk gene signature and low-risk gene signature (median survival: 13.42 months vs. 32.24 months vs. not reached, *P* < 0.001, Fig. 1B). Moreover, we also validated our model in the publicly available gastric cancer data set (GSE62254). Similar to our previous results, those GC patients with a high-risk thirty-one gene signature also had a shorter median overall survival than the patients with intermediate-risk gene signature and low-risk gene signature (median survival: 37.93 months vs. 45.77 months vs. not reached, *P* < 0.001, Supplementary Figure S).

### Gene selection by quantitative hybridization assay in GC

Since in clinical settings where reproducibility, cost, and widespread availability are key priorities, we then aimed to establish a practical prognostic algorithm based on FFPE tissue samples, we systematically measured the expression 31 genes from microarray analysis in 61 matched FFPE tissues by QGP.

For each of the 31 genes, the correlation between relative expression status by QGP and that by microarray was statistically analyzed. Twenty three genes with strong correlation between two assays were selected, including *APAF1*, *NCOA7*, *XAF1*, *IFITM1*, *EGFR*, *MMP7*, *MET*, *ERBB2*, *CDK1*, *CDK6*, *SRC*, *IGF1R*, *CDK4*, *KDR*, *FADD*, *EPHB2*, *PDCD5*, *MARCKS*, *GZF1*, *UBA2*, *MMP2*, *DYRK2*, and *B3GALT6* (Table 2). We test this 23 gene algorithm in 78 frozen microarray setting, the survival difference for each group is obvious, those high-risk gene signature GC patients had a shorter median overall survival than the patients with intermediate-risk gene signature and low-risk gene signature (median survival: 13.42 months vs. 28.95 months vs. 48.13 months, *P* < 0.001, Fig. 1C).

### The twenty-five-gene signature and survival in GC

Then we identified the gene-signature by quantitative hybridization assay. Reference genes were *TBP* and *PGK1* (Supplementary materials and methods). We establish a 25-gene (23 correlated genes plus 2 reference genes) prognostic algorithm based on FFPE tissues. And the coefficient for each of the 23 genes was derived from the previous cohort and formula of risk score calculating for each patients was changed accordingly.

In order to remove excess statistical confounding factors, patients with TNM stage IV were excluded in the training and testing cohort (the characteristics of this group is shown in Table 1). Then we evaluated this 25-gene assay in the FFPE tissue from the training cohort of 102 patients. Those high-risk GC patients were with a shorter median overall survival than the intermediate-risk and low-risk patients (median survival: 18.22 months vs. 37.02 months vs. 48.13 months, *P* = 0.017, Fig. 1D).

To confirm that the 25-gene algorithm had similar prognostic value in different populations, we tested it in an independent cohort of 360 patients. The general condition between patients in training cohort and test cohort is comparable (Table 3S). We applied the cutoff’s value for categorization in training group to the independent test set of 360 patients. Similar to the training cohort, our results showed that patients with a high-risk gene signature had a shorter median overall survival than those with a low-risk gene signature (median survival: 34.39 months vs. 37.77 months vs. not reached, *P* = 0.005) (Fig. 1E).

### Twenty-five-gene signature and survival in different TNM stages GC patients

**Figure 2:**
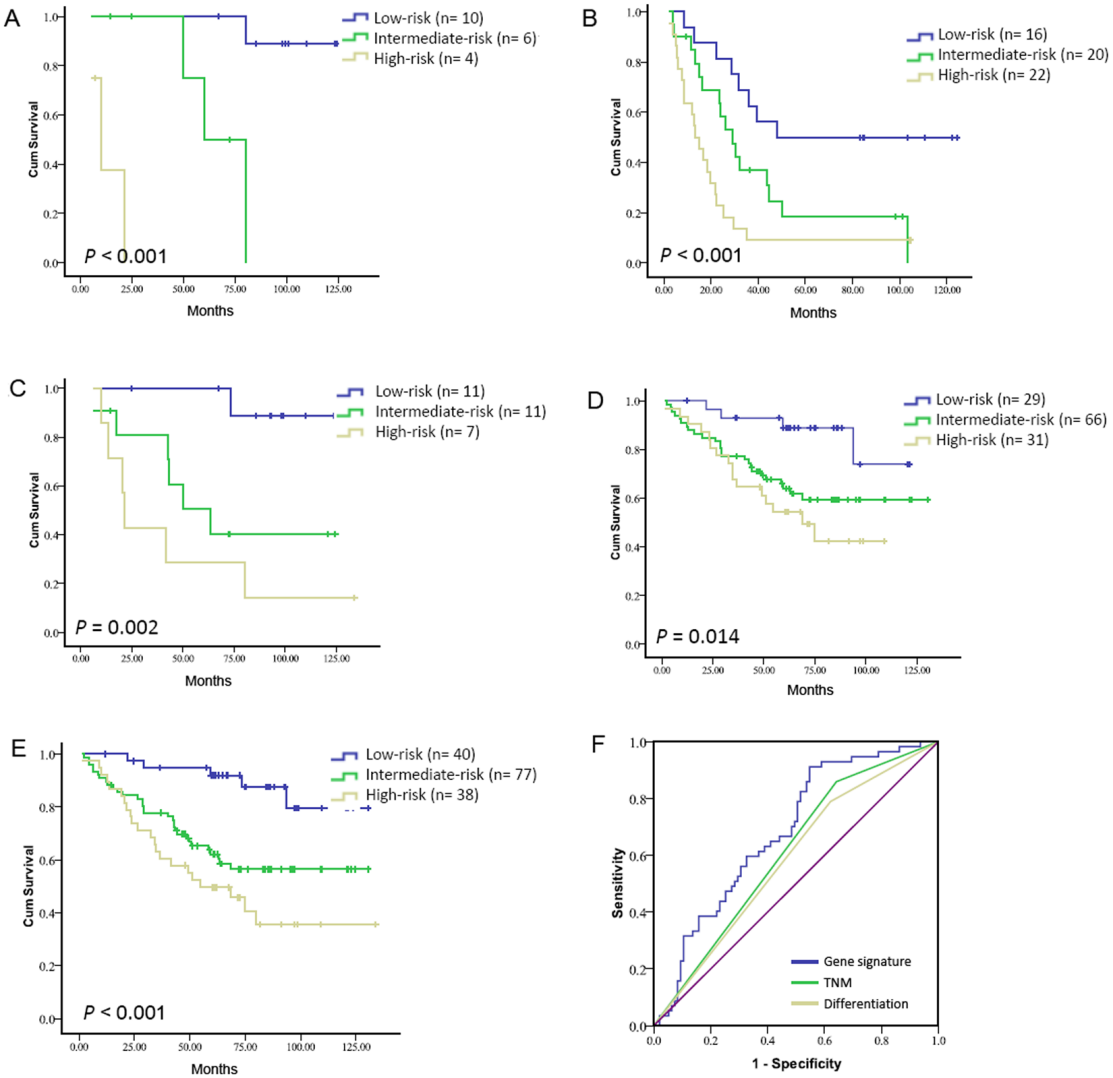
Twenty-five-gene signature and survival in GC at different TNM stages. Overall survival of patients with stage I + II and III + IV in the first cohort (**A** and **B**), stage I + II in training cohort^33^, and stage I + II in testing cohort (**D**); Panel E showed the overall survival with stage I + II disease in combined training and testing cohorts; Panel F: ROC curves compare the prognostic accuracy of the gene signature with clinicopathological risk factors in combined training and testing cohorts GC.

We also investigated the gene signature in tumor tissues obtained from GC patients with stage I and II or stage III and IV. First, we analyzed the gene signature in tumor specimens obtained from patients in the selecting cohort including 78 cases with stage I and II or stage III and IV. TNM stage I and II disease combined GC patients, those with a high-risk gene signature had a shorter overall survival than those with a low-risk gene signature (median survival: 9.97 months vs. 59.93 months vs. not reached, *P* < 0.001, Fig. 2A), among patients with stage III and IV disease, overall survival also showed similar results (median survival: 13.42 months vs. 28.95 months vs. 48.13, *P* < 0.001, Fig. 2B).

Moreover, the associations between the gene signature and prognosis in training and testing cohort were also analyzed with stage I and II or stage III respectively. In the subgroup analysis of 29 patients with TNM stage I and II of training cohort, those with a high-risk gene signature had a shorter overall survival than those with an intermediate-risk gene signature and low-risk gene signature (median survival: 21.45 months vs. 63.39 months vs. not reached, *P* **= **0.002, Fig. 2C). In the subgroup analysis of 126 patients with stage I and II disease of testing cohort, those with a high-risk gene signature also showed a shorter overall survival than those with a low-risk gene signature (median survival: 68.64 months vs. not reached vs. not reached, *P* = 0.014, Fig. 2D), while either in original training or testing cohort the overall survival in the stage III group did not differ significantly (training cohort: *P* = 0.194; testing cohort: *P* = 0.264, figure not shown).

### Twenty-five-gene assay is an independent prognostic factor in stage I and II patients

Moreover, we also noted similar results in the patients with stage I and II disease combined training and testing cohort, those with a high-risk gene signature showed a shorter overall survival than those with a low-risk gene signature (median survival: 54.57 months vs. not reached vs. not reached, *P* < 0.001, Fig. 2E).

Age, sex, gene signature, differentiation, vascular invasion, and TNM stage were included in the Cox multivariate regression analysis. According to the analysis, the high-risk gene signature, differentiation, and tumor stage II were significantly associated with death from any cause among the 155 patients (Table 3) (hazard ratio for the high-risk signature vs. the intermediate-risk signature: 5.325, 95% confidence interval, 2.061 to 13.758, *P* = 0.001; high-risk signature vs. the low-risk signature: 6.248, 95% confidence interval, 2.320 to 16.826, *P* < 0.001).

**Table 3:** Multivariate analysis of prognostic factors by Cox proportional hazard model in stage I + II GC.

Variables	Multivariate analysis	
	HR (95%CI)	*P* value
Age	1.022 (0.997–1.048)	0.087
25-gene signature		
Low-risk		
Intermediate-risk vs. low-risk	5.325 (2.061–13.758)	0.001
High-risk vs. low-risk	6.248 (2.320–16.826)	<0.001
TNM stage		
II vs. I	3.057 (1.432–6.526)	0.004
Differentiation		
Poorly vs. Well-Moderately	2.510 (1.309–4.813)	0.006

The 25-gene signature based classifier also showed significantly higher prognostic accuracy than any clinicopathological risk factor, including TNM stage and differentiation (Fig. 2F). Thus, this signature can add prognostic value to clinicopathological prognostic features.

### Twenty-five-gene signature and adjuvant chemotherapy

**Figure 3:**
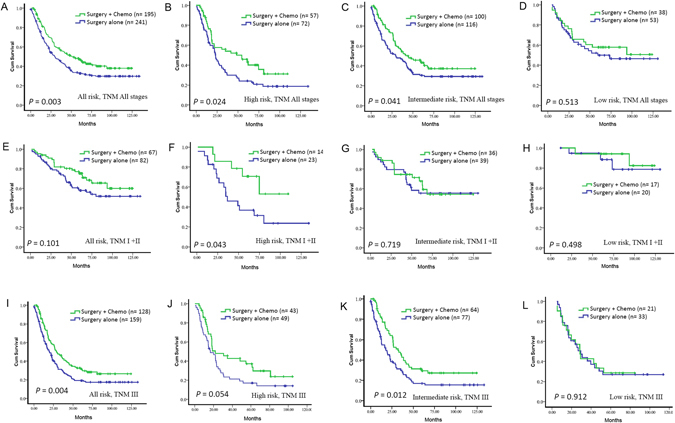
Prediction effect of chemotherapy benefit in different risk group. Kaplan-Meier survival curves for all patients (**A**–**D**), stage I + II (**E**–**H**), and III (**I**–**L**) in different-risk groups, which were stratified by the receipt of chemotherapy.

We noted adjuvant chemotherapy can enhance survival in all 436 patients (another 26 cases chemotherapy information was missed, median survival: 29.62 months vs. 50.79 months, *P* = 0.003, Fig. 3A), including high-risk median survival (median survival: 23.08 months vs. 48.42 months, *P* = 0.024, Fig. 3B), and intermediate-risk group (median survival: 29.55 months vs. 44.41 months, *P* = 0.041, Fig. 3C), while the overall survival in the patients with low-risk did not differ significantly (median survival: 59.30 vs. not reached, *P* = 0.513, Fig. 3D, Table 4S).

In the 149 stage I and II cases, adjuvant chemotherapy did not enhance survival (median survival: not reached vs. not reached, *P* = 0.101, Fig. 3E). Results from subgroup analysis using our twenty-five-gene signature based classifier showed that patients in the high-risk group had a favorable response to adjuvant chemotherapy (median survival: 36.59 months vs. not reached, *P* = 0.043, Fig. 3F). While the overall survival in the patients with either intermediate-risk or low-risk did not differ significantly (intermediate-risk group: *P* = 0.719; low-risk group: *P* = 0.498, Fig. 3G and H). In the 287 stage III cases, adjuvant chemotherapy can enhance survival (median survival: 19.99 months vs. 29.03 months, *P* = 0.004, Fig. 3I). Results from subgroup analysis using our twenty-five-gene signature based classifier showed that patients with either the high-risk or intermediate-risk had a favorable response to adjuvant chemotherapy (median survival of high-risk group, 16.54 months vs. 21.80 months, *P* = 0.054, Fig. 3J; intermediate-risk group, 17.85 months vs. 32.24 months, *P* = 0.012, Fig. 3K), while the overall survival of patients in low-risk group did not differ significantly (*P* = 0.912, Fig. 3L, Table 4S).

**Figure 4:**
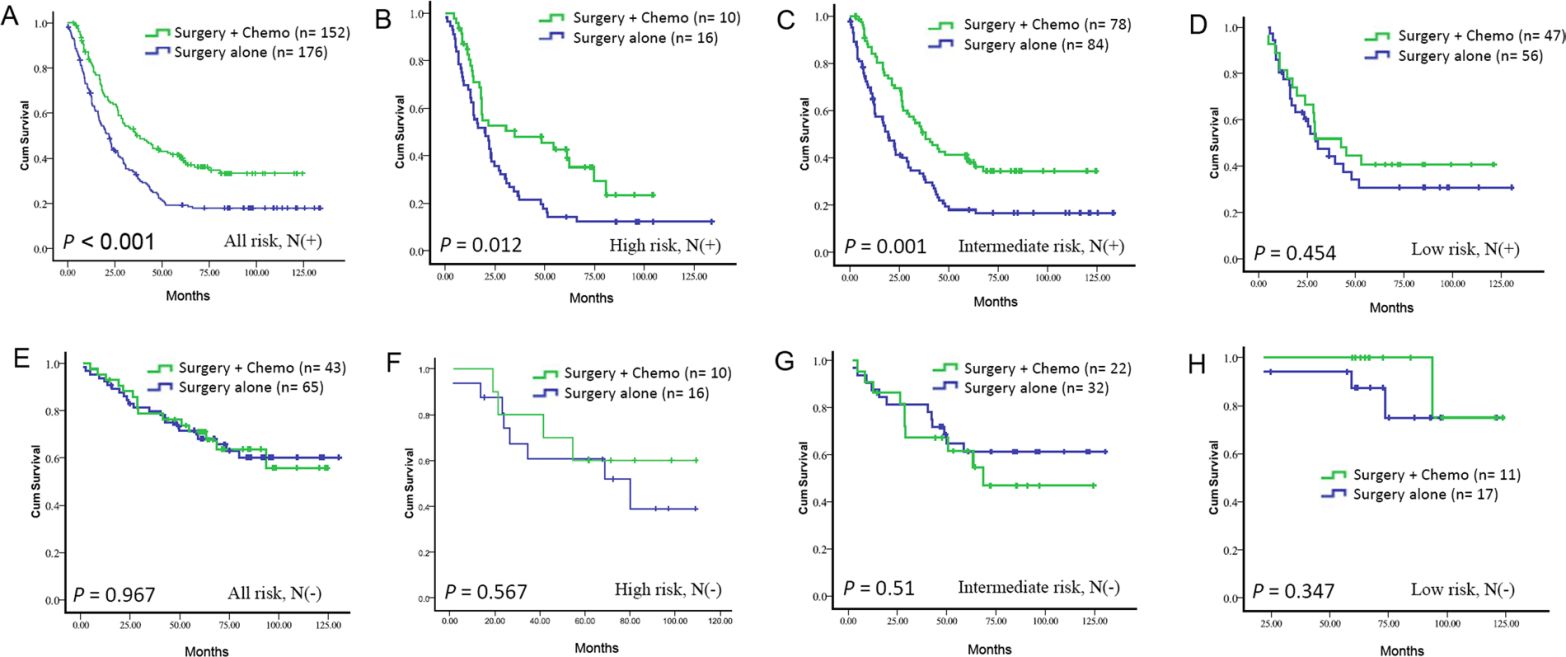
Prediction effect of chemotherapy benefit in different risk group. Kaplan-Meier survival curves for N (−) (**A–D**) and N (+) (**E–H**) in different-risk groups, which were stratified by the receipt of chemotherapy.

Moreover, the associations between adjuvant chemotherapy and prognosis in different lymph node metastasis group were also analyzed (N negative, *P* < 0.001, Fig. 4A; N positive, *P* = 0.967, Fig. 4E), we found that adjuvant chemotherapy can improve survival in GC patients with either high-risk (*P* = 0.012, Fig. 4B) or intermediate-risk (*P* = 0.001, Fig. 4C) group in lymph node metastasis positive group. In the other patients including lymph node metastasis positive group with low-risk (*P* = 0.454, Fig. 4D) and all lymph node metastasis negative group (High-risk group, *P* = 0.567, Fig. 4F; Intermediate-risk group, *P* = 0.51, Fig. 4G; Low-risk group, *P* = 0.347, Fig. 4H), overall survival is not significantly different between the chemotherapy and no chemotherapy group (Table 4S). The results indicate that our classifier could successfully identify patients who were suitable candidates for adjuvant chemotherapy.

## Discussion

Our practical, quantitative-hybridization-based assay reliably identified GC patients at high risk for mortality after surgical resection, discriminating such patients with greater accuracy than use of NCCN criteria alone. Among the twenty three genes, most of them can be generally classified into the following types: epidermal growth factor receptor, cell cycle factor, angiogenesis, matrix metalloproteinase, and apoptosis genes^17–21^. Moreover, some genes also involved in notch signaling pathway, Tor signaling pathway, regulation of transcription^22, 23^, MAPK signaling pathway, and metabolic process^24, 25^. Although other groups have developed gene signatures prognostic of survival in GC^9^, none of these previous studies used FFPE samples. Furthermore, most previous studies did not subject their prognostic signatures to large-scale, independent validation. Taken together, our assay for GC is the first of its kind in these important respects: the performance of the assay in one of the studies in a laboratory that was independent from the laboratory in which the assay was developed, the relatively large sizes of the independent testing cohorts, and the potentially large disparity between the genetic background of one of the test cohorts and that of the original training cohort used for development of the assay.

In this study, we noted that in stage I and II GC patients, those with a high-risk gene signature also showed a poor overall survival than those with a low-risk gene signature, while the overall survival of the patients with stage III did not differ significantly. This is probably because the consideration of palliative nature of surgical treatment in stage III group. The survival of patients with this stage will be affected significantly by many clinical and treatment factors other than genetic background of the cancer, and some gene classifiers study were more willing to focus on early stage cancer^26^.

Now two large Asian randomized Phase III studies (the ACTS GC and CLASSIC trials) have confirmed the survival benefit for postoperative chemotherapy after curative D2 lymph node dissection in patients with GC^27, 28^. But not all patients, especially in patients with early stage need chemotherapy and can benefit from it^29, 30^. In our study, these results indicate that our classifier could successfully identify patients with different stage who may benefit from adjuvant chemotherapy. The results indicate that the 25-gene signature could be used to select early stage GC patients at high risk for adjuvant chemotherapy and advanced stage at either intermediate or high risk for adjuvant chemotherapy. Meanwhile, it may spare early stage at either low-risk or intermediate-risk and advanced stage at low risk patients from for unnecessary chemotherapy.

In conclusion, we identified a 25-gene signature associated with prognosis in GC, and validated it in another 360 cases. Statistical analysis demonstrated it is an independent prognostic predictor. The predicting role of it in stage I and II is significantly better. Moreover, the patients with high-risk assay had a chemotherapy benefit in stage I and II GC, while low-risk patients in stage III were not found benefit from adjuvant chemotherapy.

## Materials and Methods

### Patients and samples

All of the patients with GC included in this study were diagnosed and surgically treated in Peking University Cancer Hospital between 1996 and 2007 and followed up to January 2013. This investigation was approved by the Institutional Review Boards of the hospital, informed consent was obtained from each patient, and all methods were performed in accordance with the relevant guidelines and regulations. All the frozen samples were collected and stored by Central Biobank Facility and all FFPE samples by Pathological Department of hospital. All frozen sample for this investigation passed the histological re-assessment containing at least 70% tumor cells. All FFPE tissues samples were hematoxylin-eosin (HE) stained and evaluated for one slide by two pathologists (YL and BD) and manually dissected to remove non-cancerous mucosa and mesenchymal tissues to guarantee at least 80% tumor cells. The TNM stage of GC was classified according to the 7th edition of classification recommended by the American Joint Committee on Cancer (AJCC)^31^. This investigation was performed after approval by Ethics Committee of Peking University Cancer Hospital. Informed general consent was obtained from each patient at the time of collection.

### Study design

The study design is shown in Fig. 1. At first, the frozen tissues from 78 patients (78 cancer tissues and 24 matched normal tissues) were profiled by Affymetrix Hu133Plus2 arrays for mRNA expression. Then analysis of microarray data generated candidate biomarkers related to prognosis and consensus therapeutic targets. And only biomarkers with comparable expression detected by quantitative Quantigene assay in the matched FFPE tissues were selected to develop a multiple gene assay, which was then tested and validated in two cohorts of patients with FFPE tissue samples.

In the expression profiling assay, the frozen tissues from all stages (I-IV) patient with complete clinicopathological and follow-up information were randomly selected and retrieved from the Central Biobank Facility. In the following quality control test, samples with tumor percentage <70% by histology evaluation and poor RNA quality were excluded.

In the test and validation phase, only patients with TNM stage I-III GC undergoing curative resection (histologically negative resection margin), and with complete clinicopathological and follow-up information available, were included. The reason for exclusion of stage IV from the validation cohort is because the consideration of palliative nature of surgical treatment for stage IV patients. The survival of patients with stage IV will be affected significantly by many clinical and treatment factors other than genetic background of the cancer. Patients with FFPE tissues not available or fail to pass the quality assessment were also excluded.

### Microarray Analysis

Total RNA were extracted from frozen tissues and profiled by Affymetrix Hu133Plus2 arrays for mRNA expression according to the manufacturer’s specifications. Robust Multi-array Analysis (RMA) algorithm provided by software Expression Console was used to call gene level expression values from raw signals. Based on the algorithm published, only the best probe set was selected to represent gene expressions. Any gene without probe set with informative score < 0.5 is removed from this analyses.

### Quantitative hybridization Assay in FFPE tissues

After manually dissected the FFPE slides to remove non-cancerous mucosa with scalpels, tissue homogenates were prepared according to the procedure described in the QuantiGene Sample Processing Kit for FFPE Tissues (Panomics, Inc., Fremont, CA). Briefly, 200ul of homogenizing solution supplemented with 2 µl of proteinase K (50 µg/µl) were incubated with 6 deparaffinized 5 µm sections overnight at 65 °C. Then the tissue homogenate was separated from debris by brief centrifugation, and transferred to a new tube.

Standard probe design software was used to design specific oligonucleotide probe sets for detecting target genes by QuantiGene plex 2.0 Reagent Systems (Panomics, Inc.), which gives 400-fold signal amplification. And the assay was performed according to protocols recommended by manufacturer (Panomics, Inc.). Briefly, probe set oligonucleotides were mixed with the sample solution into a 96-well plate. Target RNA was captured during an overnight incubation at 54 °C. Unbound material was removed by three washes with 200 µl of wash buffer followed by sequential hybridization of RNA amplifier molecules, then pre-amplifier hybridization, amplifier hybridization, and label probe hybridization were performed. Finally, plate were prepared for analysis after Streptavidinconjugated Phycoerythrin (SAPE) working reagent was added.

### Gene Signature and Statistical Analysis

First, the genes which showed significantly differential expression between the GC tissues and adjacent non-cancerous gastric mucosa were selected from the 78 microarray results. Then we used a Cox proportional hazards modeling as the main analytical test used to develop the prognostic algorithm. Hazard ratios from univariate Cox regression analysis were used to determine which genes were associated with death. Protective genes were defined as those associated with a hazard ratio for death of less than 1; risk genes were defined as those associated with a hazard ratio for death of more than 1. For genes that were significantly correlated with survival, we used a linear combination of the gene-expression coding values weighted by the regression coefficients to calculate a risk score for each patient. Resultant predicted risk scores from the training cohort were divided at the 33rd and 67th percentiles to generate cutoff s for categorization of risk score as low-risk, intermediate-risk, and high-risk. Kaplan–Meier analysisi was used to compare survival the survival distributions of two or more groups of a between-subjects factor with the log-rank test. Multivariate Cox proportional hazards regression analysis with backward, stepwise selection was used to evaluate independent prognostic factors associated with survival. The correlation of the microarray and QGP results was indexed by Pearson’s correlation test. *P* < 0.05 was considered to indicate statistical significance, and all tests were two-tailed.

### Significance

This study develops and validates a practical, reliable assay which can identify patients with different risk for mortality after surgery, and might be able to predict patients who may benefit from chemotherapy in GC.

## Electronic supplementary material

Supplementary Information
