# Supplementary material for: Prognostic value of a 25-gene assay in patients with gastric cancer after curative resection
Source: Sci Rep. 2017 Aug 8;7:7515. doi: 10.1038/s41598-017-07604-y (PMC5548732; doi:10.1038/s41598-017-07604-y)
Supplement: Supplementary file 1 — Supplementary Information [file 41598_2017_7604_MOESM1_ESM.pdf]

Prognostic value of a 25-gene assay in patients with gastric cancer after curative resection

Xiaohong Wang[1], Yiqiang Liu[2], Zhaojian Niu[4], Runjia Fu[3], Yongning Jia[3], Li Zhang[2], Duanfang Shao[3], Hong Du[3], Ying Hu[1], Xiaofang Xing[3], Xiaojing Cheng[3], Lin Li[3], Ting Guo[3], Ziyu Li[3], Qunsheng Ji[5], Lianhai Zhang[1, 3*], Jiafu Ji [1, 3*]

[1]Key laboratory of Carcinogenesis and Translational Research (Ministry of Education), Central Biobank Facility, [2]Department of Pathology, and [3]Department of Surgery, Peking University Cancer Hospital and Institute, Beijing, China; [4]Department of General Surgery, Affiliated Hospital of Qingdao University, Shandong Province, China; [5]Asia & Emerging Markets Innovative Medicine, AstraZeneca R&D, Shanghai, China

X. Wang, Y. Liu, Z. Niu and R. Fu contributed equally to this work as co-first authors.

Corresponding Authors: L. Zhang (zlhzlh@hotmail.com) or J. Ji (jijiafu@hsc.pku.edu.cn). Department of Surgery, Peking University Cancer Hospital and Institute, Beijing, China, 100142; Phone numbers: +86 10 88196319

Supplemental materials and methods

Risk Score Calculation

The regression coefficients of 31 genes were as follows: CDK1, 0.053; IGF1R, 0.199; MET, 0.044; EGFR, 0.103; FADD, 0.38; MARCKS, 0.65; DYRK2, 0.7; SRC, 0.122; PDCD5, 0.317; B3GALT6, 0.815; CDK4, 0.265; ERBB2, 0.04; EPHB2, 0.288; MTOR, 1.05; KDR, 0.35; GZF1, 0.615; MMP2, 0.003; FLT4, 1.231; ITCH, 0.732; ERBB3, 0.03; CDK6, 0.167; UBA2, 0.496; NCOA7, -0.554; XAF1, -0.559; CDK3, -0.447; MMP7, -0.008; APAF1, -1.065; IFITM1, -0.408; TCF7L2, -1.001; PARP1, -0.316; PDGFRB, -0.038. Then a patient's risk score was derived by a summation of each gene expression level times its corresponding coefficient as follows: Risk score = (0.053 ×CDK1 value) + (0.199 × IGF1R value) + (0.044 × MET value) + (0.103 × EGFR value) + (0.38 × FADD value) + (0.65 × MARCKS value) + (0.7 × DYRK2 value) + (0.122 × SRC value) + (0.317 × PDCD5 value) + (0.815 × B3GALT6 value) + (0.265 × CDK4 value) + (0.288 × EPHB2 value) - (1.05 × MTOR value) + (0.35 × KDR value) + (0.615 × GZF1 value) + (0.003 × MMP2 value) + (1.231 × FLT4 value) + (0.732 × ITCH value) + (0.03 × ERBB3 value) + (0.167 × CDK6 value) + (0.496 × UBA2 value) - (0.554 × NCOA7 value)- (0.559 × XAF1 value)- (0.447 × CDK3 value) - (1.065 × APAF1 value) - (0.408 × IFITM1 value) - (1.001 × TCF7L2 value) - (0.316 × PARP1 value) - (0.038 × PDGFRB value). The risk score was used to classify patients

into high, intermediate or low risk signature, in which a high risk score indicated a poorer survival for patients. To avoid the effect of extreme values and set the number of patients in the three groups (high vs. intermediate vs. low risk signature) equal in the training dataset, the 33th, 67th percentile (median) were chosen as the cut-off value. The regression coefficients were applied directly to the 23 gene signature. In the testing dataset, both the regression coefficients of risk score and the cut-off value derived from the training dataset were applied directly where risk score = (0.053 ×CDK1 value) + (0.199 × IGF1R value) + (0.044 × MET value) + (0.103 × EGFR value) + (0.38 × FADD value) + (0.65 × MARCKS value) + (0.7 × DYRK2 value) + (0.122 × SRC value) + (0.317 × PDCD5 value) + (0.815 × B3GALT6 value) + (0.265 × CDK4 value) + (0.288 × EPHB2 value) - (1.05 × MTOR value) + (0.35 × KDR value) + (0.615 × GZF1 value) + (0.003 × MMP2 value) + (1.231 × FLT4 value) + (0.732 × ITCH value) + (0.03 × ERBB3 value) + (0.167 × CDK6 value) + (0.496 × UBA2 value) - (0.554 × NCOA7 value)- (0.559 × XAF1 value)- (0.447 × CDK3 value) - (1.065 × APAF1 value) - (0.408 × IFITM1 value) - (1.001 × TCF7L2 value) - (0.316 × PARP1 value) - (0.038 × PDGFRB value).

Selection of reference genes

First, ABI TaqMan Human Endogenous Control Plate were used to select the reference

genes in GC, including RPLPO, PPIA, PGK1, HMBS, TBP, GAPDH, IPO8, UBC, HPRT1, POLR2A, B2M, YHWAZ, GUSB, TFRC, ACTB, and 18S. Our results showed that the stability of PGK1， B2M， TBP， UBC， PPIA gene is well (Figure 1). Further using these gene as reference genes in QGP analysis, we found that PGK1 and TBP had high stability and abundance (Figure 2, 3).

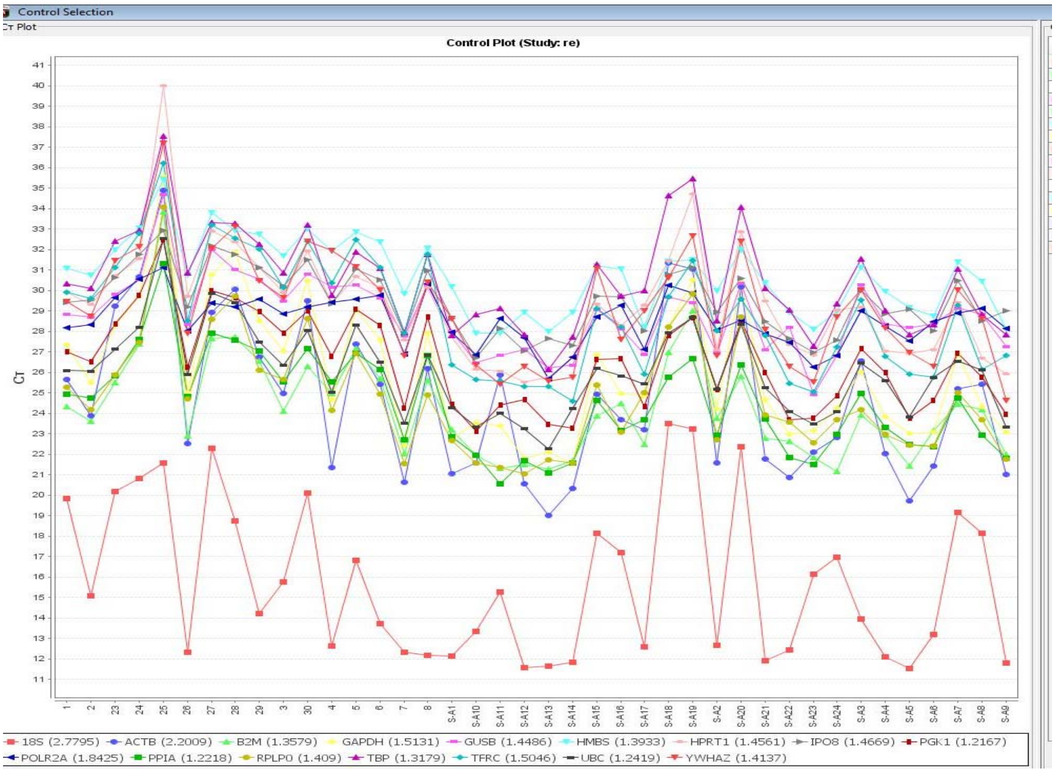

Figure 1 Gene selection by ABI TaqMan Human Endogenous Control Plate

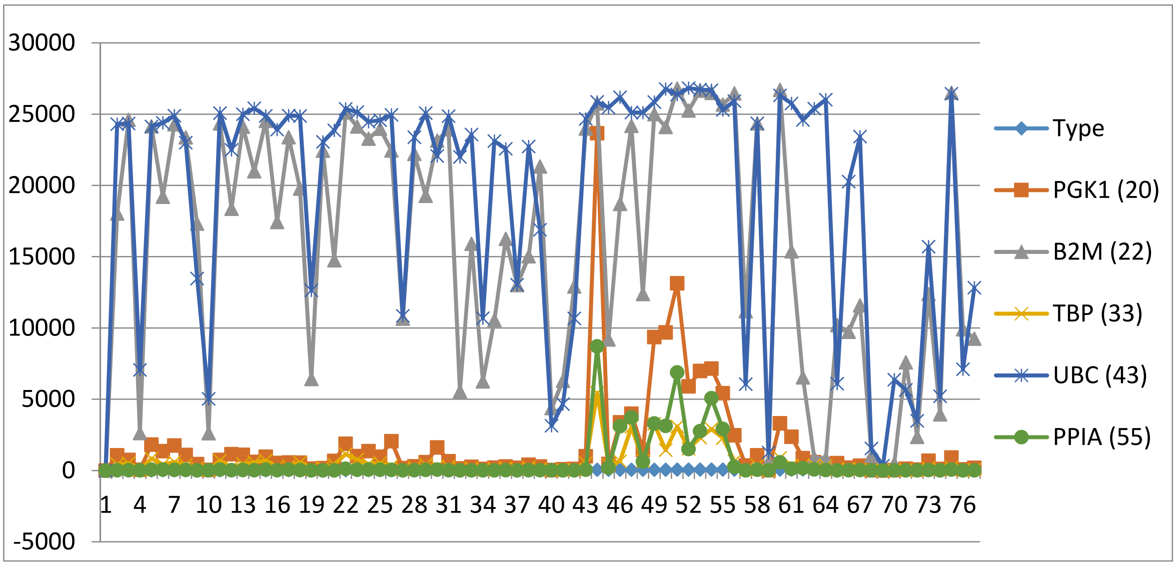

Figure 2 The stability and abundance of PGK1， B2M， TBP， UBC， PPIA gene

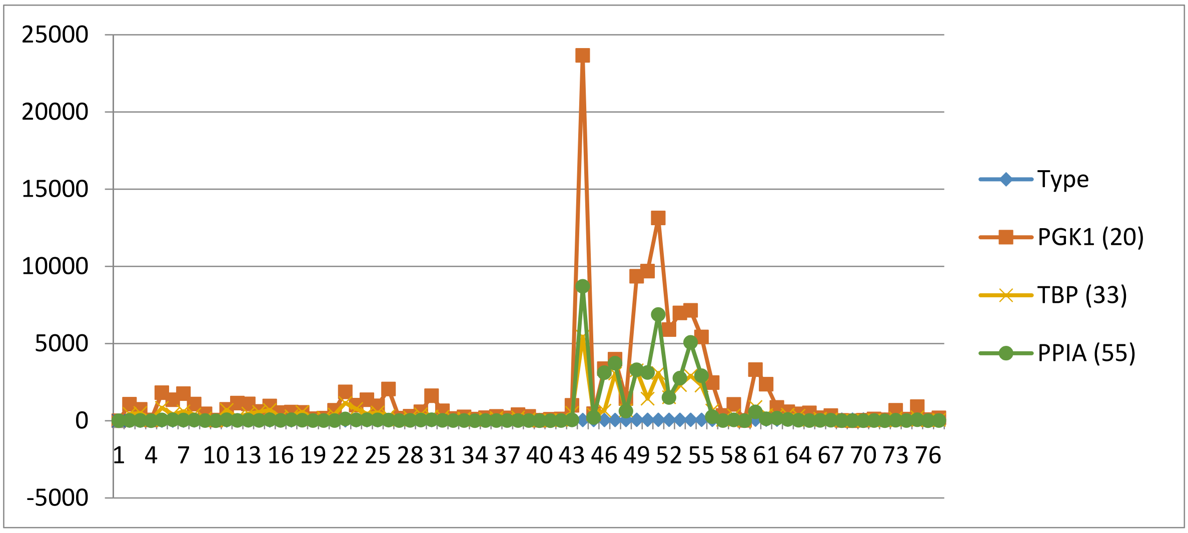

Figure 3 The stability and abundance of PGK1， PPIA， TBP gene

Figure S

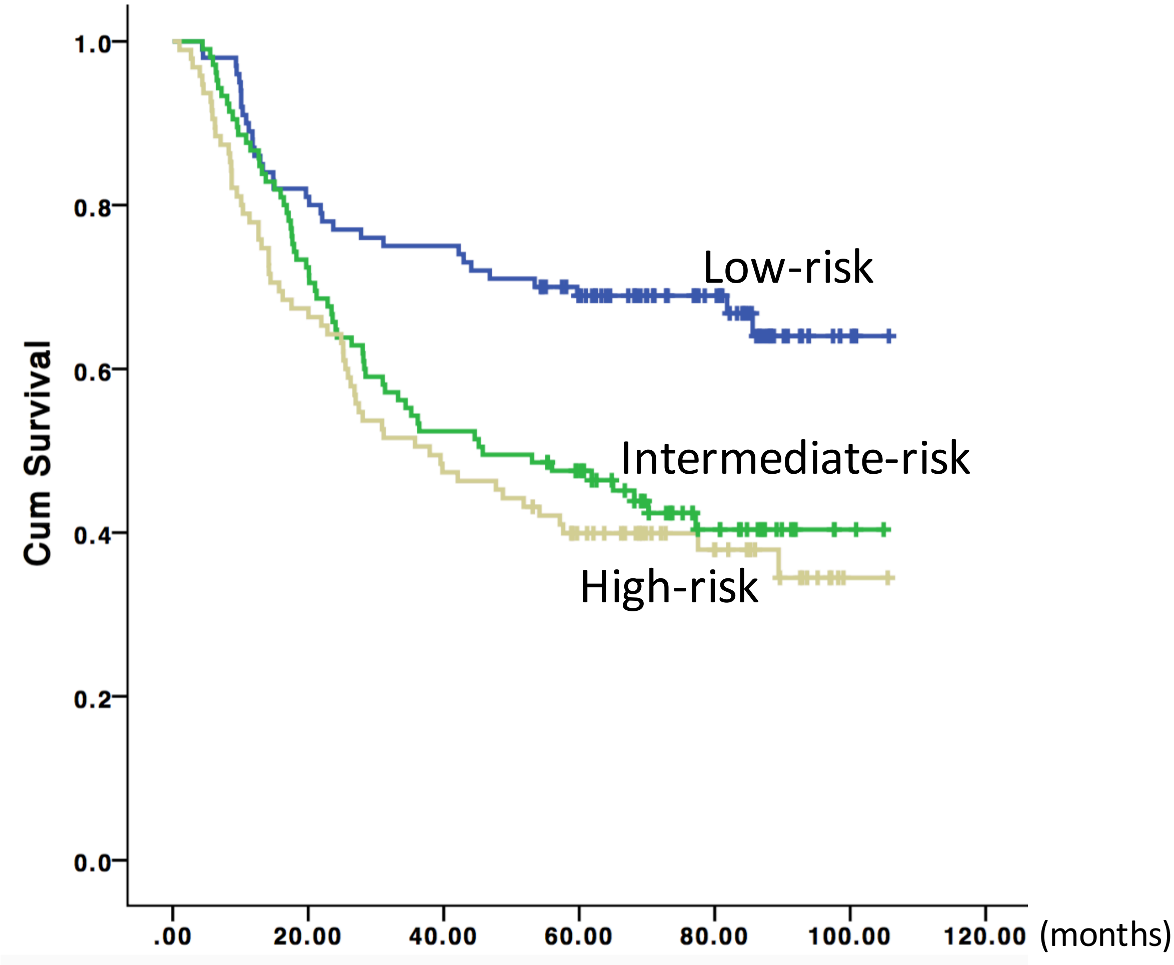

The gene signature and survival in GSE62254 data set
